# Exposure of adipocytes to bisphenol-A *in vitro* interferes with insulin action without enhancing adipogenesis

**DOI:** 10.1371/journal.pone.0201122

**Published:** 2018-08-22

**Authors:** Elena De Filippis, Ting Li, Evan David Rosen

**Affiliations:** 1 Division of Endocrinology, Beth Israel Deaconess Medical Center and Harvard Medical School, Boston, Massachusetts, United States of America; 2 HEAL^th^ Program, Mayo Clinic, Scottsdale, Arizona, United States of America; Universidad Miguel Hernandez de Elche, SPAIN

## Abstract

Bisphenol-A (BPA) is a lipophilic compound widely used in the manufacture of plastic items and thought to play a role in the growing obesity epidemic. Recent publications suggest that BPA may have a pro-adipogenic effect. Here we explore the effect of low, but environmentally relevant, concentrations of BPA on adipogenesis using a variety of cellular models. Mouse 3T3-L1, C3H10T1/2 and human adipose-derived stromal cells (hADSCs) were cultured with BPA concentrations ranging from 0.1nM to 100μM. We failed to observe positive effects on differentiation at any dose or in any model. 3T3-L1 adipocytes differentiated with high concentrations of BPA showed decreased mRNA expression of several adipocyte markers. Mature adipocytes differentiated in the presence of BPA were insulin resistant, with an approximate 25% reduction in insulin-stimulated glucose uptake. This was accompanied by a significant decrease in insulin-stimulated Akt phosphorylation, and an increase in mRNA levels of inflammatory markers (i.e. IL-6, TNFα). In conclusion, low, but environmentally relevant, doses of BPA may contribute to the development of a chronic, low-grade inflammatory state in exposed adipocytes, which in turn may affect adipose tissue insulin sensitivity, independent of adipogenesis. These studies suggest an alternative mechanism by which BPA may contribute to the development of obesity.

## Introduction

In 1962 Rachel Carson published “Silent Spring” bringing the negative, profound effects that low doses of man-made compounds have on animals, the ecosystem and the environment to public attention [1]. This observation sparked numerous scientific projects investigating how chemicals can affect hormonal physiology and/or homeostasis in both humans and animals. More than 50 years later, the Endocrine Society released the first Scientific Statement summarizing the most significant evidence collected from clinical, epidemiological, and basic research demonstrating that some chemicals pose a significant risks to public health [2]. However, despite significant effort, much remains unclear about the mechanisms by which man-made chemicals exert their negative effects on human health [2, 3].

There is excellent evidence that EDCs can affect male and female reproduction, breast development and cancer, neuroendocrinology, prostate cancer and thyroid function [4]. Recently, it has been suggested that EDCs might also play a role in the obesity epidemic and type 2 diabetes mellitus [5, 6]. Accordingly, the “obesogen hypothesis” proposes that environmental toxins may promote weight gain by facilitating fat storage in existing fat cells (hypertrophy), and/or may promote *de novo* adipogenesis (hyperplasia) [7, 8]. EDCs may favor caloric storage over utilization, or they could alter hormonal control of appetite and satiety [9].

Among the many EDCs identified, bisphenol-A (BPA), a small molecule commonly used in the production of polycarbonate plastics [10], is the most prevalent. BPA is used in the production of food and water containers, dental sealant and many other household items; significant levels have been found in human serum, urine, and breast milk [11–14]. Animal studies have shown that elevated levels of BPA can induce abnormalities in sperm and oocyte maturation thereby disrupting fertility, and can also affect immune function [4]. More recently, it has been observed that perinatal exposure of mice to BPA increases adipose tissue mass, raises serum cholesterol level and may predispose to the development of metabolic syndrome [15–17]. A series of studies conducted in animal models highlighted that exposure to BPA in either adult animals [6, 18] or *in utero* [17] leads to the development of insulin resistance. Epidemiologic studies (reviewed in [3]) have identified an association between high urinary level of BPA, and development of obesity, insulin resistance, and diabetes. In cultured pre-adipocyte cell lines, treatment with BPA ranging between 0–100μg/ml for two days early in differentiation was reported to accelerate differentiation [19, 20] via a PI3K-dependent pathway. Enhanced adipogenesis was observed in cells treated with either BPA alone or in combination with 5 μg/ml of insulin. Of note, the BPA concentrations used in these studies were very high, raising the question of what effect, if any, would be seen at levels that approximate those seen in humans. One recent study did look at the effect of lower concentrations of BPA (1nM) delivered throughout the two weeks differentiation process in 3T3-L1; this treatment induced enhanced lipid droplet accumulation [21].

In the present study we sought to determine whether adipogenesis, or other properties of adipocytes, could be perturbed by exposure to BPA, using a variety of rodent and human cellular models.

## Material and methods

### Cell culture

3T3-L1 pre-adipocytes (American Type Culture Collection) were grown to confluence in Dulbecco’s modified Eagle’s medium (DMEM; Invitrogen) with 10% bovine calf serum (BCS; Hyclone) in 5% CO_2_. Two days after confluence, to achieve optimal differentiation, cells were exposed to DMEM/10% fetal bovine serum (FBS; Invitrogen) with 1μM dexamethasone (Sigma), 4μg/ml insulin (Sigma), and 0.5mM isobutylmethylxanthine (Sigma) for 48hrs. For suboptimal differentiation, only dexamethasone and insulin were added for 48 hrs. After 2 days, cells were maintained in medium containing FBS only.

C3H10T1/2 cells (American Type Culture Collection) were grown to confluence in Minimum Essential Media (MEM: Gibco) with 10% fetal bovine serum in 5% CO_2_. For experiments requiring suboptimal differentiation conditions, 1μM dexamethasone (Sigma), 4μg/ml insulin (Sigma), and 0.5mM isobutylmethylxanthine (Sigma) were added upon confluence for 48 hours. This resulted in 30% of confluent cells reaching full differentiation as determined by Oil Red O staining.

Human adipose derived stem cells obtained from samples of subcutaneous liposuction of obese volunteers[22] were differentiated using a protocol modified from [23]. Briefly, hADSCs were grown in MesenPRO RS medium (Invitrogen) plus 4ng/ml FGF-2 on plates coated with 10μg/cm^2^ collagen I (Invitrogen). To achieve suboptimal differentiation conditions, two days after confluence cells were placed in induction medium (DMEM/F12 supplemented with 10% FBS plus 1μM dexamethasone, 1.7μM insulin, 0.5mM IBMX, 5μM rosiglitazone, 17μM pantothenic acid, and 33μM biotin). 72 hours later, the medium was replaced with maintenance medium (DMEM/F12 with 10% FBS plus 1μM dexamethasone, 17μM pantothenic acid, and 33μM biotin); media was replaced every two days thereafter.

For all BPA (Sigma) dose curve and time course experiments, BPA was dissolved in 0.1% ethanol and added at the concentration (0.1nM to 100μM) and time (day-2 to 2 or d-2 to 7).

### Lipid staining of the cells, Oil Red O

Cells were fixed with 4% Formaldehyde-Fresh (Fisher Scientific) for 15 min at room temperature and stained with oil red O solution (0.5% oil red O in isopropyl alcohol/water = 3:2) for 2 h. Cells were washed twice with distilled water before photography.

### Analysis of gene expression by qPCR

Total RNA was extracted from tissues with TRIzol reagent (Invitrogen) according to the manufacturer’s instructions. 1μg total RNA was converted into first-strand cDNA using the RETROscript first strand synthesis kit as described by the manufacturer (Ambion Inc). Quantitative PCR (qPCR) was performed using an 7900 HT Fast Real-Time PCR System (Applied Biosystems) with specific primers (Table 1) and SYBR Green PCR Master Mix (Life Technologies). The relative abundance of mRNAs was standardized with β-actin mRNA as the invariant control.

**Table 1:** Primers used for RT-PCR analysis.

Gene ID		Primer Sequence (5’ to 3’)
**PPARG**	**Forward**	CAAGAATACCAAAGTGCGATCAA
	**Reverse**	GAGCTGGGTCTTTTCAGAATAATAAG
**CEBPA**	**Forward**	GACCATTAGCCTTGTGTGTTAC
	**Reverse**	TGGATCGATTGTGCTTCAAGTT
**FABP4**	**Forward**	CTTCAAACTGGGCGTGGAA
	**Reverse**	CTAGGGTTATGATGCTCTTCACCTT
**FASN**	**Forward**	GTTTTGAGGGATGCCATGCT
	**Reverse**	GGGTTGCCCTGTCAAGGTT
**LPL**	**Forward**	ACAAAGTGTTCCATTACCAAGTCAAG
	**Reverse**	GTGCCGTACAGAGAAATTTCGA
**ADIPOQ**	**Forward**	CAGTGGATCTGACGACACCAA
	**Reverse**	CTGGGCAGGATTAAGAGGAACA
**TNFa**	**Forward**	CCCTCACACTCAGATCATCTTCT
	**Reverse**	GCTACGACGTGGGCTACAG
**IL-1B**	**Forward**	AAATACCTGTGGCCTTGGGC
	**Reverse**	CTTGGGATCCACACTCTCCAG
**IL-6**	**Forward**	CCGTGTGGTTACATCTACCCT
	**Reverse**	CGTGGTTCTGTTGATGACAGTG
**NR3C2**	**Forward**	CTTGAGTTGGAGATCGTACAAACATAC
	**Reverse**	TCATACATGGCAGACTGATGCA
**IL-13**	**Forward**	AGACCAGACTCCCCTGTGCA
	**Reverse**	TGGGTCCTGTAGATGGCATTG
**B-ACTIN**	**Forward**	CCTGAGGCTCTTTTCCAGCC
	**Reverse**	TAGAGGTCTTTACGGATGTCAACGT
**36B4**	**Forward**	GAGGAATCAGATGAGGATATGGGA
	**Reverse**	AAGCAGGCTGACTTGGTTGC

### Glucose uptake assay

Mature 3T3-L1 adipocytes in 24-well plates were washed twice with Krebs-Ringer (KR) phosphate buffer (127mmol/L NaCl, 4.7mmol/L KCl, 0.9mmol/L MgSO_4_, 10mmol/L NaPO_4_, 0.9mmol/L CaCl_2_) and incubated with pre-warmed KR phosphate buffer containing 0.2% fatty acid–free BSA and 100nM insulin. The dish was then placed at 37°C for 30 min. After this period, ^3^H-2-deoxyglucose and unlabeled 2-deoxyglucose were dispensed into each well for a final concentration of 1uCi/mL and 0.1mmol/L, respectively. Cells were incubated for an additional 5 min at 37°C, and the reaction was stopped by adding ice-cold (KR) phosphate buffer with 200mM D-(+)-Glucose with 10uM Cytochalasin B. Lysis buffer was applied to each well, incubated for 30 min at room temperature, then lysates were transferred to scintillation vials to be counted in a β-counter (Beckman).

### Western blotting

Cell lysates were prepared in RIPA buffer with MiniComplete Protease Inhibitor mixture (Roche) unless described otherwise. Total protein was quantified using the DC method (Bio-Rad), and all lysates were treated with Laemmli buffer and boiled for 5 min at 95°C. Twenty micrograms of protein lysate was resolved by 10% SDS/PAGE and transferred onto PVDF membranes. Membranes were blocked in PBS supplemented with 0.5% Tween 20 (PBST) plus 10% nonfat milk for 1 h followed by incubation overnight in primary rabbit anti-phospho-(p)-Akt (Cell Signaling Technology, Cat#4060), anti-Akt (Cell Signaling Technology, Cat#4691), primary mouse anti-Insulin receptor (IR) β (Cell Signaling Technology, Cat#3020), primary rabbit anti-phospho-(p)-IRβ (Cell Signaling Technology, Cat#3024), primary rabbit anti-phospho-(p)-IRS1 (Cell Signaling Technology, Cat#2386), primary rabbit anti-IRS1 (Cell Signaling Technology, Cat#2383). The blots were then washed with TBS-T and incubated in secondary anti-rabbit antibody (Jackson ImmunoResearch Lab., Inc Cat# 111-035-003) or in secondary anti-mouse antibody (Jackson ImmunoResearch Lab., Inc Cat# 115-035-003) for 1 h. The blots were rinsed in TBS-T and incubated in SuperSignal West Pico Chemiluminescent substrate (Pierce) before being exposed to film. All blots were quantified using ImageQuantTL software.

#### Statistical analysis

All experiments were performed as multiple biological replicates (at least triplicate), and results are expressed as mean ± SEM. Statistical significance was determined by comparing results from BPA exposed and non-exposed cells using the Student’s *t* test using in the Microsoft Excel statistical package. A probability value of *p* < 0.05 was considered statistically significant.

## Results

### High doses of BPA do not enhance adipogenesis in 3T3-L1 pre-adipocytes

Previously published data [19] demonstrated that confluent 3T3-L1 pre-adipocytes treated with 20μg/ml (87μM) BPA for 48 hours accumulated a greater number of lipid droplets compared to vehicle treatment. Increased accumulation of lipid was also described after prolonged BPA treatment throughout the time course of differentiation (from day -2 to day 8) [19]. In our hands, we did not observe enhanced lipid accumulation under the same experimental conditions (S1 Fig). As a result, we decided to adopt a protocol where we could test several different doses of BPA (1nM to 1μM) for varying lengths of exposure, reasoning that this would allow us to broadly investigate whether BPA affects adipogenesis in 3T3-L1 cells.

**Fig 1:**
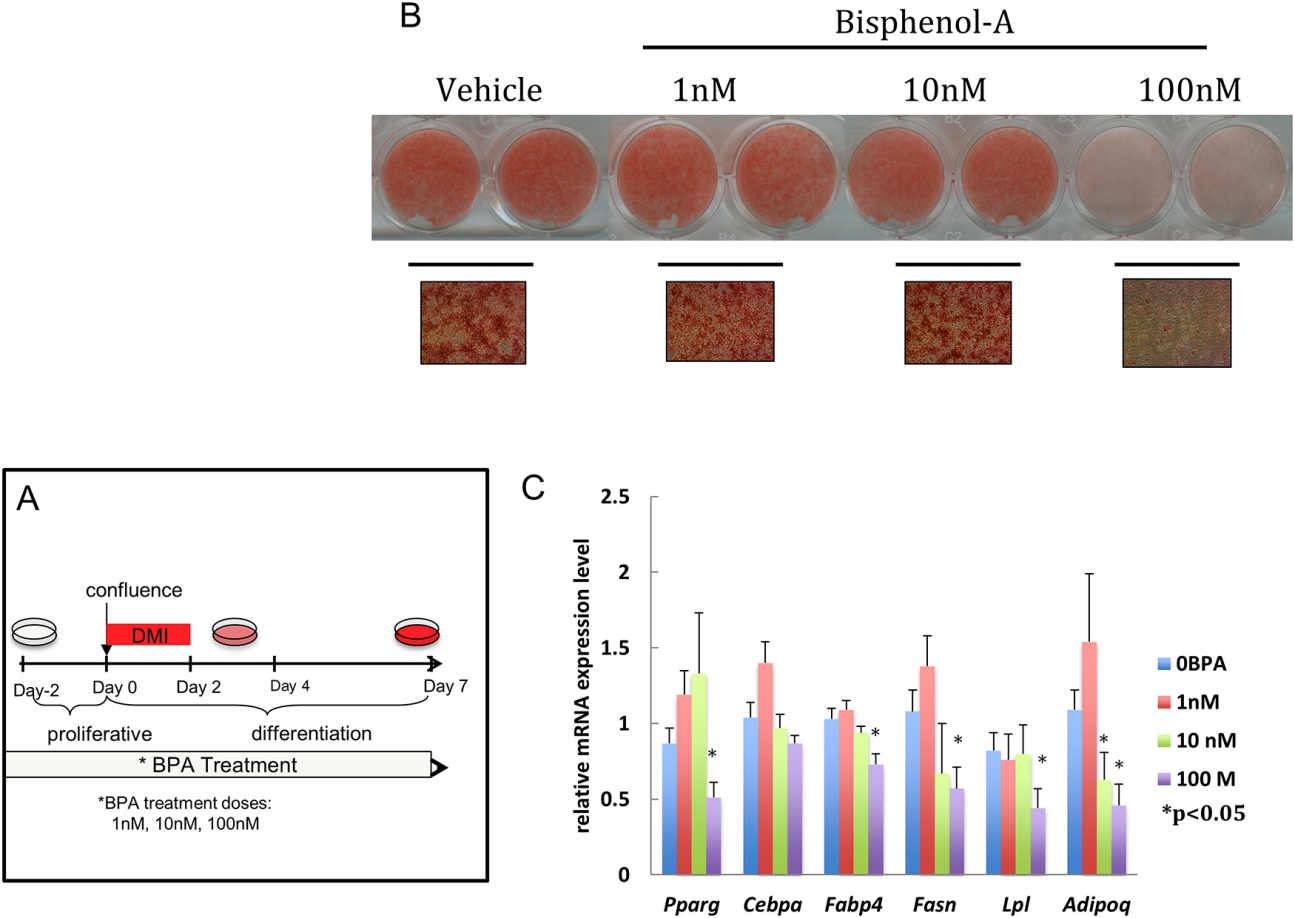
Effect of continuous BPA treatment on adipogenesis. Confluent 3T3-L1 cells were exposed to different concentrations of BPA (1-100nM) or Vehicle (0.1% ethanol) from day -2 to day 7. Adipocyte differentiation was induced at day 0 with media enriched with dexamethasone, insulin and isobutylmethylxanthine (DMI). (A) Schematic representation of the treatment dose and time course applied. (B) Triglyceride accumulation was visualized by oil Red O staining and representative bright field microscopy images (40X magnification) were acquired between day 8–9. (C) Quantitative reverse transcription PCR (qRT-PCR) analysis of adipocyte marker gene expression was performed between day 8–9. Gene expression was normalized to 36B4 and is presented as relative mRNA expression level. *p<0.05 versus vehicle treated cells exposed to same protocol.

Exposure of differentiating adipocytes (days -2 to 7) stimulated with canonical differentiating conditions (DMI: Dexamethasone, 3-isobutyl-1-methylxanthine, insulin) to doses of BPA ranging from 1nM to 100nM failed to show increased accumulation of lipid droplets when compared to vehicle (Fig 1). Interestingly, we noted that a 100nM dose had an inhibitory effect on differentiation (Fig 1B). Consistent with the lipid staining, BPA treatment did not increase levels of any of the tested adipocyte marker genes, while 100nM actually reduced some mRNA levels (e.g. *Pparg*, *Fabp4*, *Fasn*) (Fig 1C).

**Fig 2:**
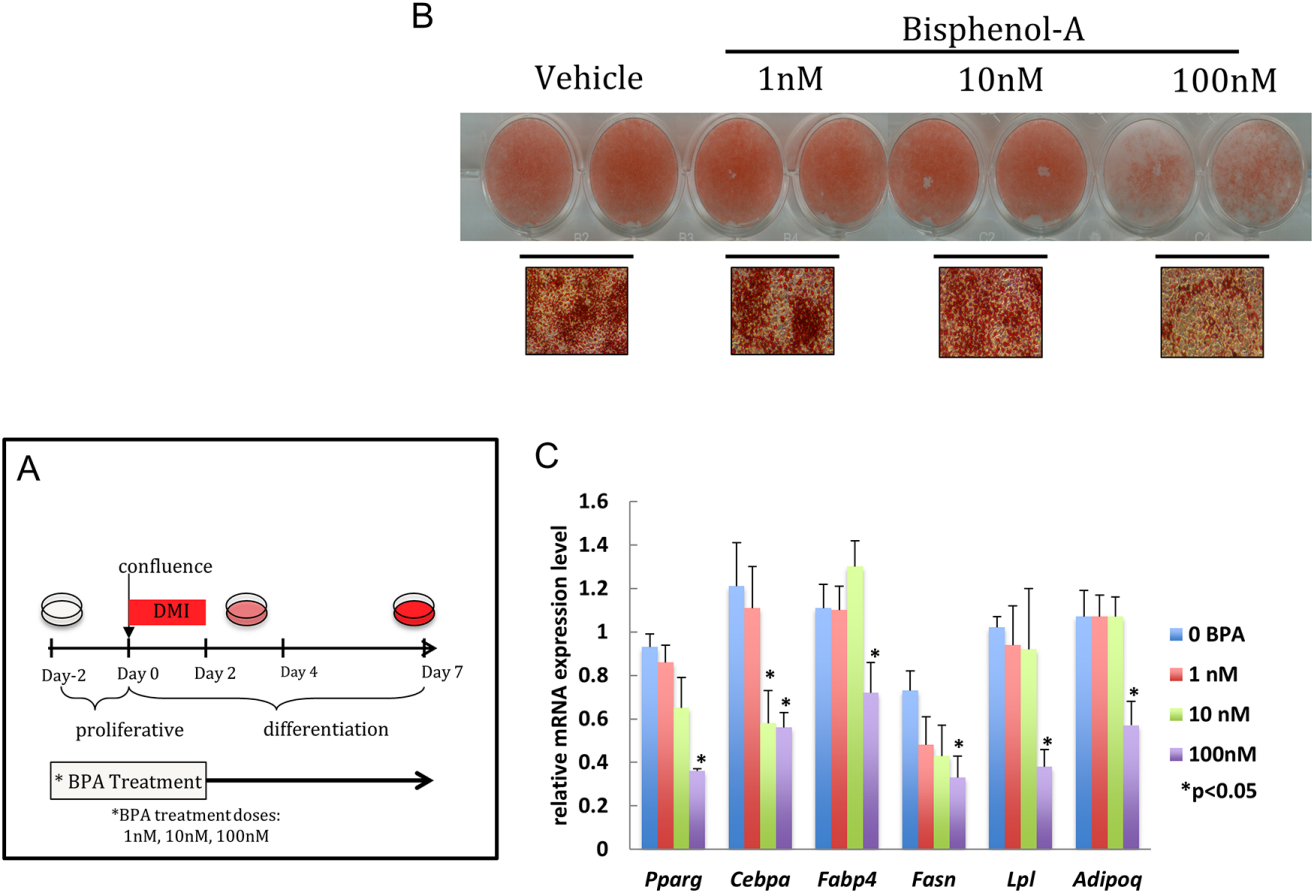
High doses of BPA during early adipogenesis inhibits differentiation. Confluent 3T3-L1 cells were exposed to different concentrations of BPA (1-100nM) or vehicle (0.1% ethanol) from day -2 to day 2. Adipocyte differentiation was induced at day 0 with media enriched with DMI. (A) Schematic representation of the treatment dose and time course applied. (B) Triglyceride accumulation visualized by oil Red O staining and representative bright field microscopy images (40X magnification) were acquired between day 8–9. (C) Quantitative reverse transcription PCR (qRT-PCR) analysis of adipocyte marker gene expression was performed between day 8–9. Gene expression was normalized to 36B4 and is presented as relative mRNA expression level. *p<0.05 versus vehicle treated cells exposed to same protocol.

The inhibitory effect of high doses of BPA (100nM) was also evident when BPA was added for shorter period of time during a critical phase of the differentiation process (day -2 to 2) (Fig 2B and 2C). Reducing the duration of exposure to BPA (day -2 to day 0 or day 0 to day 2) failed to show any significant change in oil red O staining, defining a minimal critical period of BPA exposure in this model (S2 Fig).

### Low doses of BPA fail to accelerate adipocyte differentiation

**Fig 3:**
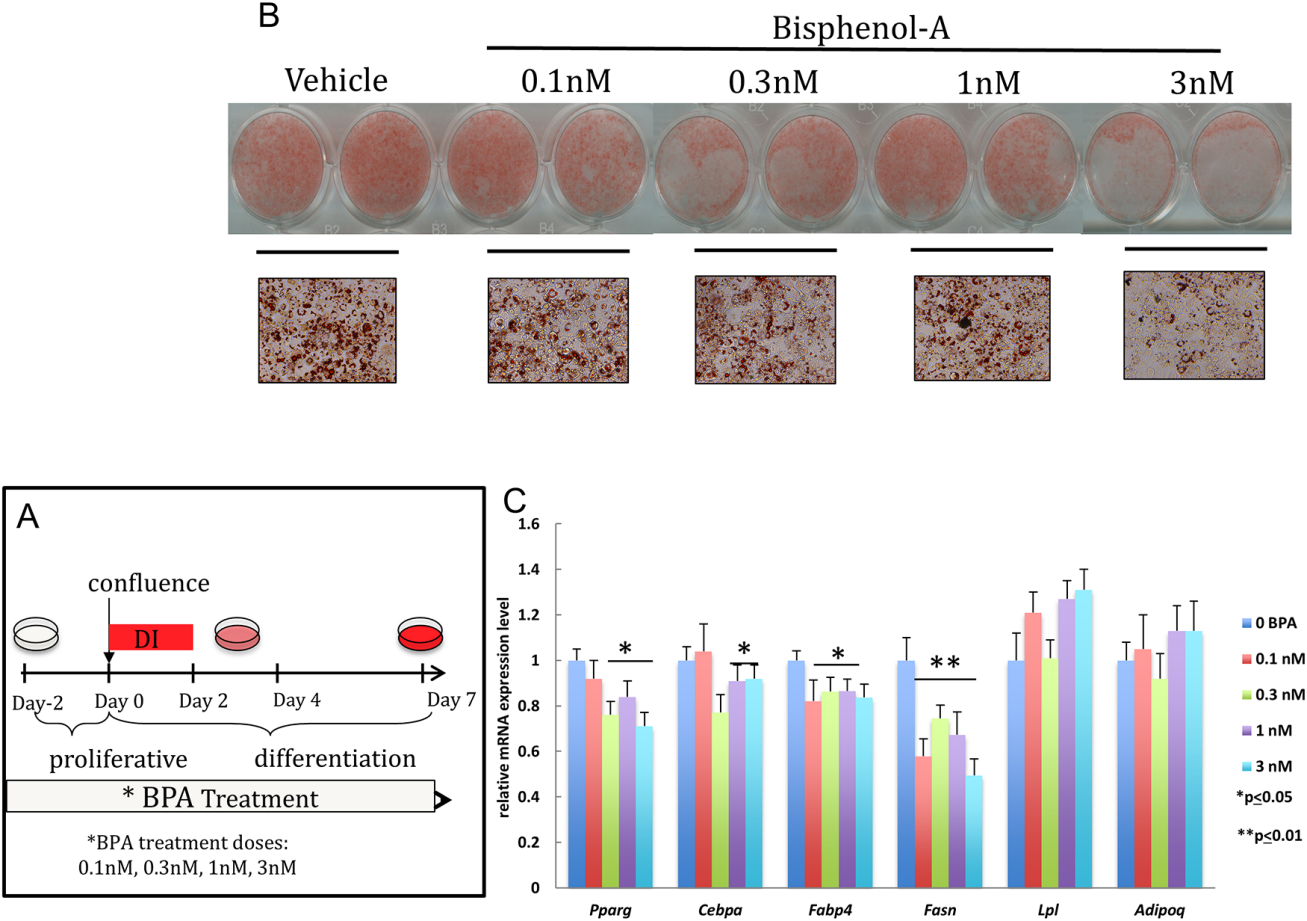
Environmentally relevant doses of BPA applied throughout differentiation inhibit adipogenesis. Confluent 3T3-L1 pre-adipocytes were exposed to different concentrations of BPA (0.1-3nM) or vehicle (0.1% ethanol) from day -2 to day 7. Suboptimal adipocyte differentiation was induced at day 0 with media enriched with dexamethasone and insulin (DI). (A) Schematic representation of the treatment dose and time course applied. (B) Triglyceride accumulation visualized by oil Red O staining and representative bright field microscopy images (40X magnification) were acquired between day 8–9. (C) Quantitative reverse transcription PCR (qRT-PCR) analysis of adipocyte marker gene expression was performed between day 8–9. Gene expression was normalized to 36B4 and is presented as relative mRNA expression level. *p≤ 0.05,**p≤0.01 versus vehicle treated cells exposed to same protocol.

BPA has been reported known to exhibit a non-monotonic dose response (NMDR) [10, 24], meaning that lower concentrations of this compound can paradoxically elicit an increased cellular response. This, combined with reports in the literature of low concentrations detected in biological samples from human cohorts (fat, serum, and milk) [25–27], led us to test the effect of lower, environmentally relevant concentrations of BPA. To this end, we exposed differentiating 3T3-L1 pre-adipocytes to concentrations of BPA ranging from 0.1nM to 3nM, for the full course of differentiation (days -2 to 7) (Fig 3). In addition, we chose to adopt suboptimal conditions for differentiation by adding only dexamethasone and IBMX (DI) to confluent cells, reasoning that this strategy would better highlight potential pro-adipogenic actions of BPA. Overall, adipogenesis was not significantly accelerated compared to vehicle-only treated cells (Fig 3B and 3C), and BPA concentrations of 1nM and 3nM negatively affected gene expression (e.g. *Pparg*, *Cebpa*, *Fabp4*, *Fasn*). Shorter periods of BPA exposure (day-2 to 2) also failed to increase adipogenesis (S3 Fig).

### BPA exposure does not affect cellular commitment to the adipose lineage

During the early stages of adipogenesis, mesenchymal stem cells (MSCs) become committed to the adipose lineage, restricting them from becoming myoblasts, chondrocytes or osteoblasts [28]. Following commitment, subsequent exposure to adipogenic stimuli triggers terminal differentiation. 3T3-L1 and 3T3-F442A cells are *in vitro* models of committed pre-adipocytes, and thus they cannot be used to assess the effects of BPA or other perturbagens on the commitment process. To address this, we studied murine C3H10T1/2 and human adipose-derived stem cells (hADSCs), which represent examples of uncommitted cell systems.

**Fig 4:**
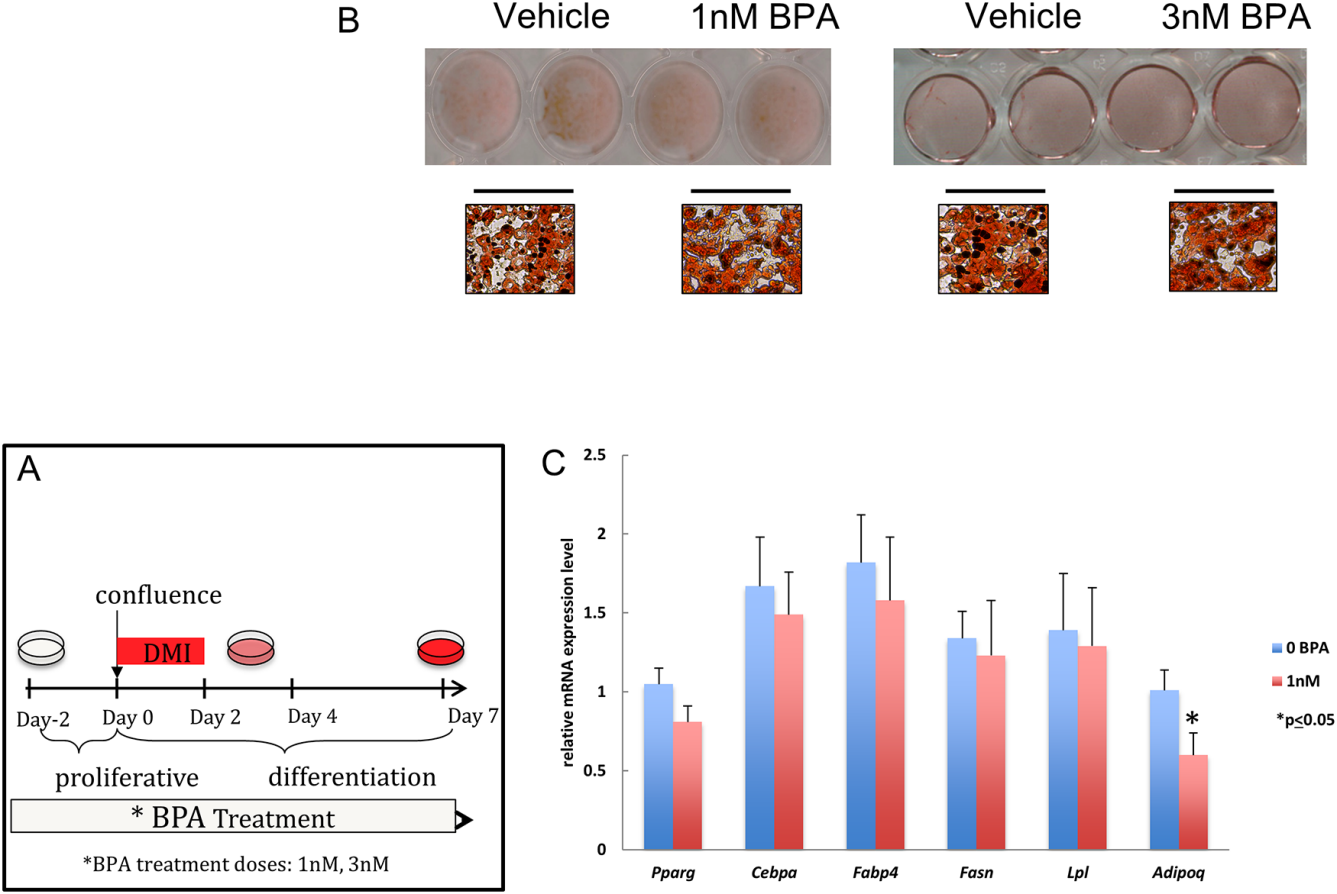
Adipogenesis in C3H10T1/2 cells is unchanged in presence of BPA treatment. Confluent C3H10T1/2 cells were exposed to either 1nM or 3nM of BPA or vehicle (0.1% ethanol) from day -2 to day 7. Suboptimal adipocyte differentiation was induced at day 0 with media enriched only with DMI. (A) Schematic representation of the treatment dose and time course applied. (B) Triglyceride accumulation visualized by oil Red O staining and representative bright field microscopy images (40X magnification) were acquired between day 8–9. (C) Quantitative reverse transcription PCR (qRT-PCR) analysis of adipocyte marker gene expression was performed between d8-d9 only on the 1nM BPA treatment. Gene expression was normalized to 36B4 level and is presented as relative mRNA expression. *p≤ 0.05 versus vehicle treated cells exposed to same protocol.

We tested 2 doses of BPA (1nM and 3nM) during either the full differentiation process (day -2 to 7) or restricted to a smaller window from day -2 to 2. C3H10T1/2 (Fig 4B and 4C; S4B and S4C Fig) or hADSCs (S5 Fig) were stimulated with suboptimal adipogenic conditions (SC) in order to better assess whether, in presence of BPA, adipogenesis was affected. We were unable to detect any effect of BPA at any dose or duration in either of these models, involving either oil Red O accumulation (S4 and S5 Figs) or mRNA gene expression (Fig 4C).

In summary, low, but environmentally relevant BPA doses did not increase adipogenesis in either committed or pre-committed cellular systems.

### BPA affects insulin action in mature adipocytes

**Fig 5:**
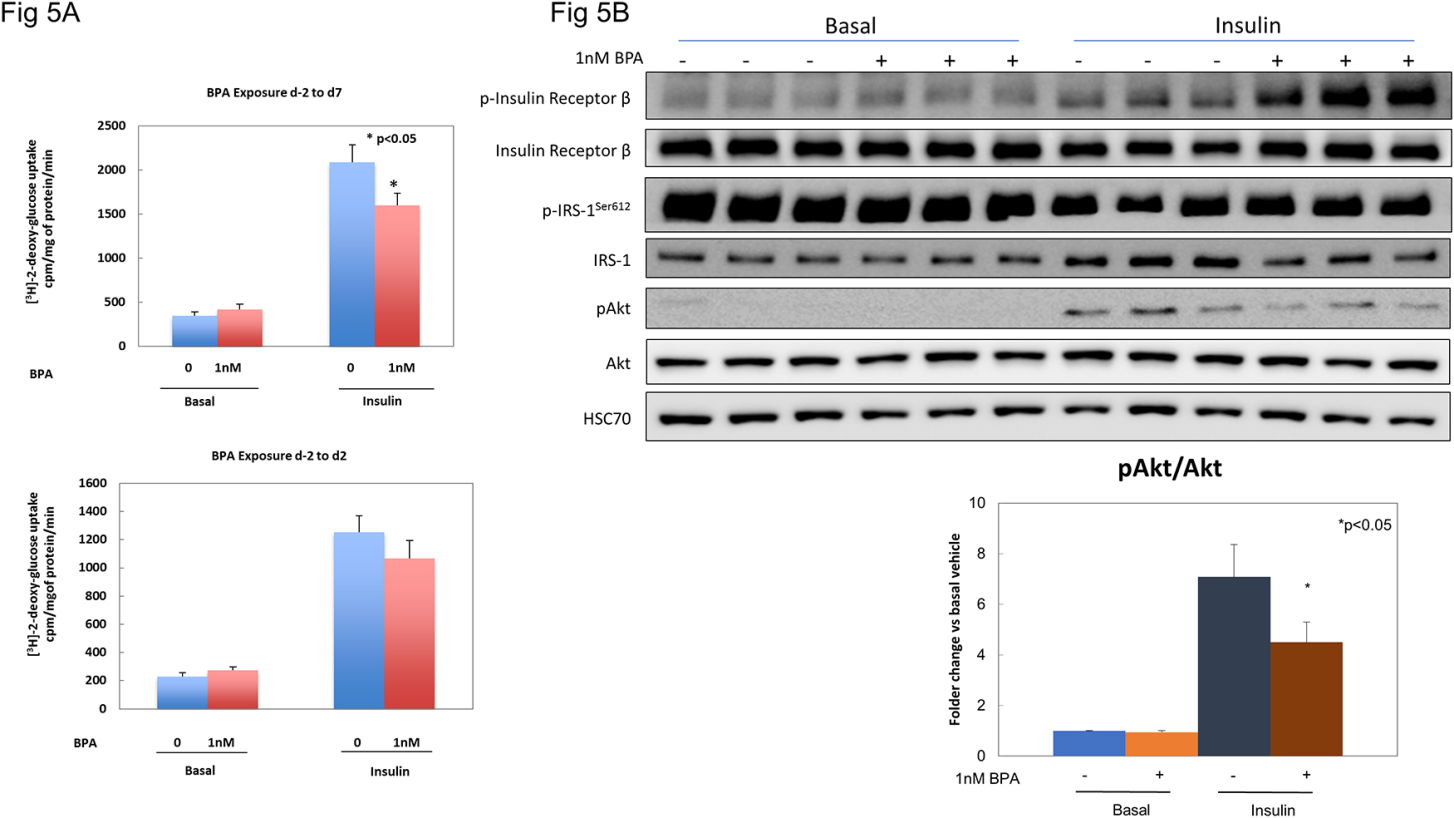
Reduced insulin-stimulated glucose uptake and insulin signaling in mature adipocytes exposed to low doses of BPA during differentiation. (A) Basal and insulin-stimulated glucose uptake in 3T3-L1 cells exposed to 1nM BPA from day -2 to day 7 (upper panel) or day -2 to day 2 (lower panel). n = 3 independent experiments, each point is the mean of 6 experimental replicates, *p<0.05 versus untreated cells. (B) Immunoblotting selected insulin-signaling pathway related protein in 3T3-L1 cell lysate under basal and insulin stimulated conditions. Quantification expressed as pAkt/Akt fold change vs basal, non-treated cells. *P<0.05 vs non-treated. Insulin dose 100nM.

We next assessed whether exposure to a low concentration of BPA during differentiation could affect the function of mature adipocytes. 3T3-L1 pre-adipocytes were differentiated in the presence of 1nM BPA from day -2 to day 7. These cells, or vehicle treated controls, were then assessed for their ability to take up glucose in response to insulin. We noted no difference in basal glucose uptake, but insulin was less effective at promoting glucose uptake in BPA-treated cells (Fig 5A). This was associated with a significant decrease in insulin-stimulated Akt phosphorylation (Fig 5B), but no other changes in the tested insulin signaling pathway, including pIRS1/IRS1 ratio. Shorter exposure to BPA led to a trend toward diminished insulin-stimulated glucose uptake that did not reach significance (Fig 5A, **right panel**).

### BPA causes mature adipocytes to increases mRNA levels of pro-inflammatory markers

**Fig 6:**
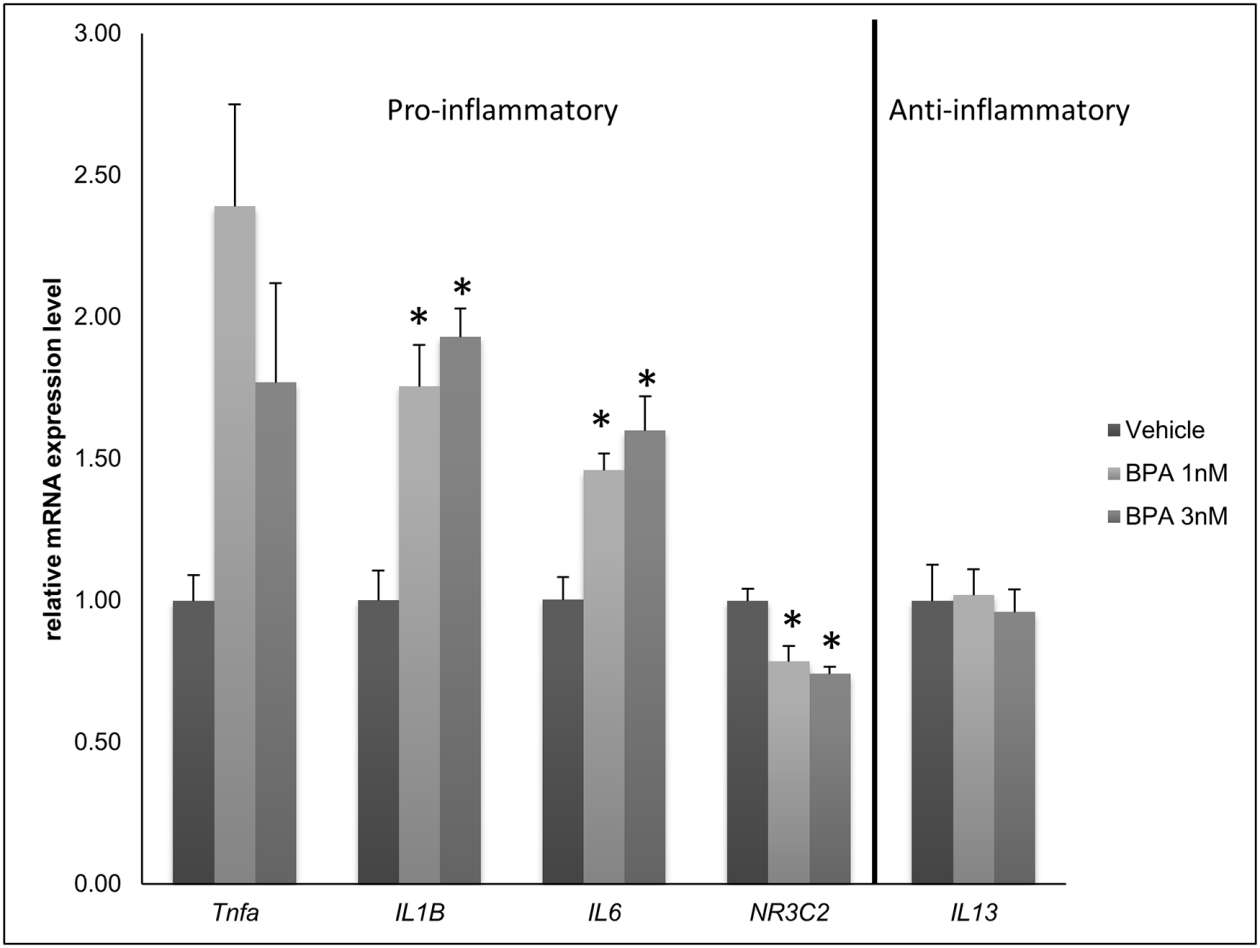
Altered mRNA levels of inflammatory markers in mature adipocytes exposed to low doses of BPA during differentiation. Confluent 3T3-L1 pre-adipocytes were exposed to different doses of BPA (1nM or 3nM) or vehicle (0.1% ethanol) from day -2 to day 7. Quantitative reverse transcription PCR (qRT-PCR) analysis of inflammatory markers Tumor Necrosis Factor-α (Tnfa), Interleukin-1β (IL-1B), Interleukin-6 (IL-6) and Nuclear receptor subfamily3, group C, member 2 (NR3C2) and Interleukin-13 (IL13) anti-inflammatory gene expression was performed between day 8–9. Gene expression was normalized to β-Actin and is presented as relative mRNA expression level. *p<0.05 versus vehicle treated cells exposed to same protocol.

Adipose tissue insulin resistance is intimately related to increased local inflammation [29]. We therefore evaluated whether low concentrations of BPA (1nM; 3nM) during the full-time course of adipogenesis (days -2 to 7) would increase mRNA inflammatory markers. TNF-α and IL-6 were both significantly increased, compared to vehicle (Fig 6).

## Discussion

BPA has been suggested to contribute to the obesity epidemic via an ability to enhance adipogenesis. One early report showed that 3T3-L1 pre-adipocytes exposed to high concentrations of BPA (20μg/ml or 87μM) either alone or in combination with insulin was sufficient to accelerate the conversion to mature adipocytes [19]. The authors concluded that prolonged exposure to BPA in vivo could lead to the development of obesity by increasing overall adipocyte formation.

After this initial report, others have supported the idea that BPA could enhance adipogenesis. For example, Sargis and colleagues [30] treated 3T3-L1 cells with various EDCs and found that picomolar BPA concentrations could increase adipogenesis via a GR-dependent mechanism. Similarly, Boucher and colleagues [31] evaluated the effect of BPA (1–50μM) treatment on primary human preadipocytes. The authors were able to show a small increase in post-differentiation lipid accumulation by oil Red O staining after 14 days exposure to BPA at two different concentrations. This increase was significant compared to vehicle treatment only, but much less than the increase achieved by dexamethasone stimulation. Adipocyte marker mRNA levels were also minimally elevated in the presence of BPA. Expression of these markers was inhibited when BPA was added together with estrogen receptor antagonist such as ICI, suggesting this effect is mediated by non-classical ER pathways.

In the present study, we show that low, environmentally relevant concentrations of BPA failed to accelerate adipogenesis in a variety of cellular systems. In fact, we observed significantly reduced adipocyte-specific gene expression at both high and low doses of BPA, especially when the latter exposure took place early in the process of terminal differentiation.

It is not immediately apparent why our results differ than some of the previously published findings. In some (but not all) cases, some the differences may be explained by the use of more refined techniques to assess adipogenesis (QPCR vs LPL activity and DNA content). Furthermore, some authors used very high doses of BPA, bringing pathophysiologic relevance into question. Review of the human literature for BPA levels in fluids or tissue is on the order of ng/ml or nM [25–27].

We chose to use a suboptimal cocktail of differentiation (omitting IBMX) to better appreciate any potential effect of BPA on the adipogenic process. Even in this setting, we were still unable to detect an appreciable positive effect of BPA on adipogenesis. When we shortened the length of exposure to BPA (limiting to day -2 to 2) we identified negative effects on differentiation, indicating that exposure during some critical early window is an important time for interaction between BPA and the adipogenic machinery.

In this study we evaluated the effect of BPA to affect adipogenesis using multiple *in vitro* cellular systems, including human ADSCs. Our data are consistent with those from Valentino and colleagues, who also showed no effect of BPA on differentiation of hADSCs [32]. Based on these results we conclude that chronic exposure of fat tissue to BPA is unlikely to be a driver in the expansion of fat tissue, at least as mediated through direct adipocyte differentiation.

Interestingly, Valentino et al. demonstrated that BPA exposure (for up to 48hrs) leads to decreased glucose uptake (measured as residual glucose concentration in the medium before and after insulin exposure) and increased cytokine secretion in hADSCs[33]. In a more recent study, Ariemma et al., exposed 3T3-L1 preadipocytes to 1nM of BPA for two weeks prior induction of adipogenesis. In contrast with our findings, the authors report an increase in lipid accumulation upon stimulation of adipogenesis, while demonstrating an increase in mRNA levels of inflammatory markers (IL6, INFγ). In our experimental model we expose preadipocytes only for 48hrs to BPA prior to induction of adipogenesis and continue BPA treatment until day of analysis. The difference between these two studies may potentially reside in the window of treatment adopted by Ariemma et al. [21]. The timing of preadipocyte exposure may not play a fundamental role on the induction of inflammatory markers or impairment of the insulin signaling, as shown by the authors on evaluation of insulin signaling at day 0. In our experimental model, we chose to expose differentiating adipocytes to BPA (day-2 to day7), putting less emphasis on the preadipocyte component of the exposure (only 48hrs). In accordance with the data presented by Ariemma et al., we confirmed that exposure of low doses BPA during differentiation may contribute to generate dysfunctional adipocytes where a reduced insulin-stimulated glucose uptake and Akt phosphorylation associates with an increases mRNA levels of pro-inflammatory cytokines such as TNF-α and IL-6, without affecting expression of adiponectin. This leads us to speculate that *in vivo*, chronic exposure to BPA may contribute to a chronic, low grade inflammatory state which, in turn, may affect adipose tissue insulin sensitivity. This observation, together with the previously cited work in animal models [6, 17, 18], suggests that BPA may be a more potent diabetogenic agent than an obesogenic one. Additional animal studies are required to prove this hypothesis, but if found to be true, could implicate BPA as a player in the development of insulin resistance and Type 2 diabetes, without causing the development of obesity.

Indeed, it should be pointed out that there is an underlying logical fallacy in assuming that increased adipogenesis could be a driver of obesity. Obesity represents the storage of excess calories and derives ultimately from an imbalance between calories taken in and calories expended [34]. There is often an increase in adipogenesis in response to overnutrition, but this should not be conflated to a role as a cause of obesity; rather, the body is attempting to protect itself from the toxic effects of overnutrition by generating more safe receptacles for excess calories. Consistent with this idea, animals and humans with reduced adipogenesis have significant metabolic dysfunction, including insulin resistance, hepatic steatosis, and glucose intolerance [35]. In light of this, our observations that BPA may alter the *quality* of adipocyte differentiation such that they are predisposed toward insulin resistance and inflammation likely has significantly more biological importance than effects on the *quantity* of differentiation per se.

## Supporting information

S1 Fig BPA alone or in combination with insulin fails to induce adipogenesis. Confluent 3T3-L1 were exposed to 20μg/ml of BPA or vehicle (0.1% ethanol) from day -2 to day 2 or from day-2 to day 7. Adipocyte differentiation was induced at day 0 with media enriched with either 5μg/ml of insulin or with DMI. (Left Panel) Schematic representation of the treatment concentrations and time course applied. (Right Panels) Triglyceride accumulation visualized by oil Red O staining and representative bright field microscopy images (40X magnification) were acquired between day 8–9.
(TIF)

S2 Fig High doses of BPA in addition to DMI do not enhance adipogenesis. Confluent 3T3-L1 were exposed to several concentrations of BPA (1-100nM) or vehicle (0.1% ethanol) from day -2 to day 0 or from day 0 to day 2. Adipocyte differentiation was induced at day 0 with media enriched with DMI. (Left Panels) Schematic representation of the treatment concentrations and time course applied. (Right Panels) Triglyceride accumulation visualized by oil Red O staining and representative bright field microscopy images (40X magnification) were acquired between day 8–9.
(TIF)

S3 Fig Low doses of BPA in addition to suboptimal differentiation conditions do not accelerate adipogenesis. Confluent 3T3-L1 cells were exposed to several doses of BPA (0.1-3nM) or vehicle (0.1% ethanol) from day -2 to day 2. Suboptimal adipocyte differentiation was induced at day 0 with media enriched with only with DI (as detailed in the *Methods*). (A) Schematic representation of the treatment dose and time course applied. (B) Triglyceride accumulation visualized by Oil Red O staining and representative bright field microscopy images (40X magnification) were acquired between day 8–9. (C) Quantitative reverse transcription PCR (qRT-PCR) analysis of adipocyte marker gene expression was completed between day 8–9. Gene expression was normalized to 36B4 level and is presented as relative mRNA expression.
(TIF)

S4 Fig Low doses of BPA in addition to suboptimal differentiation media does not accelerate adipogenesis in pre-Committed cells. Confluent C3H10T1/2 cells were exposed to either 1nM or 3nM of BPA or vehicle (0.1% ethanol) from day -2 to day 2. Suboptimal adipocyte differentiation was induced at day 0 with media enriched with only with DMI (as detailed in the *Methods*). (A) Schematic representation of the treatment dose and time course applied. (B & C) Triglyceride accumulation visualized by oil Red O staining and representative bright field microscopy images (40X magnification) were acquired between day 8–9.
(TIF)

S5 Fig Exposure of differentiating hADSCs to low doses of BPA does not enhance adipogenesis. Confluent hADSCs were exposed to several concentrations of BPA (1-3nM) or vehicle (0.1% ethanol) from day -2 to day 7 or from day -2 to day 2. Adipocyte differentiation was induced at day 0 with media enriched with suboptimal conditions (SC) (as detailed in the *Methods* section). (Left Panels) Schematic representation of the treatment concentrations and time course applied. (Right Panels) Triglyceride accumulation visualized by oil Red O staining and representative bright field microscopy images (40X magnification) were acquired between day 8–9.
(TIF)
