# Supplementary figures and images for: Exposure of adipocytes to bisphenol-A in vitro interferes with insulin action without enhancing adipogenesis
Source: PLoS One. 2018 Aug 22;13(8):e0201122. doi: 10.1371/journal.pone.0201122 (PMC6104924; doi:10.1371/journal.pone.0201122)

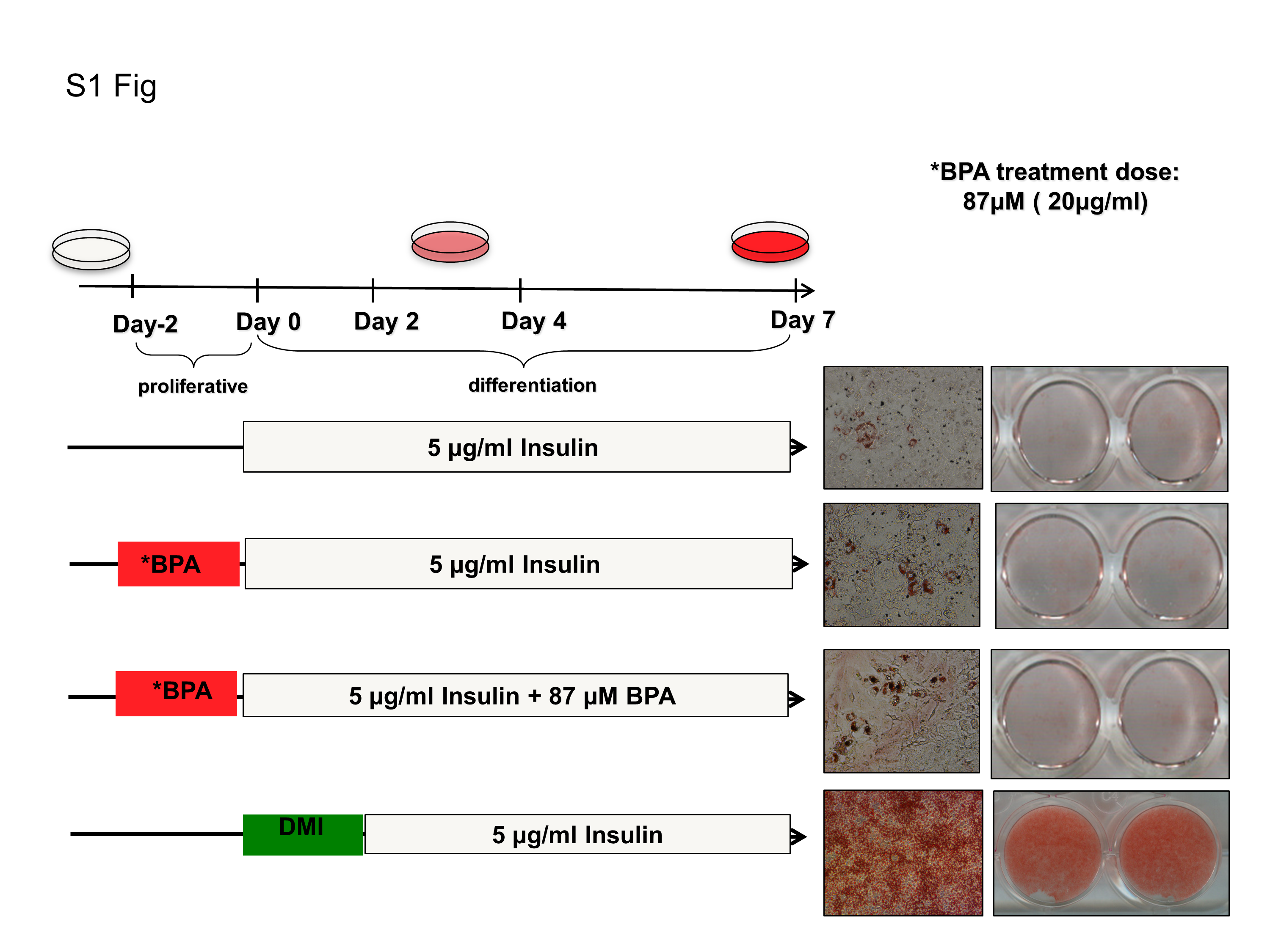

Supplement: S1 Fig — Confluent 3T3-L1 were exposed to 20μg/ml of BPA or vehicle (0.1% ethanol) from day -2 to day 2 or from day-2 to day 7. Adipocyte differentiation was induced at day 0 with media enriched with either 5μg/ml of insulin or with DMI. (Left Panel) Schematic representation of the treatment concentrations and time course applied. (Right Panels) Triglyceride accumulation visualized by oil Red O staining and representative bright field microscopy images (40X magnification) were acquired between day 8–9. (TIF) [file pone.0201122.s001.TIF]

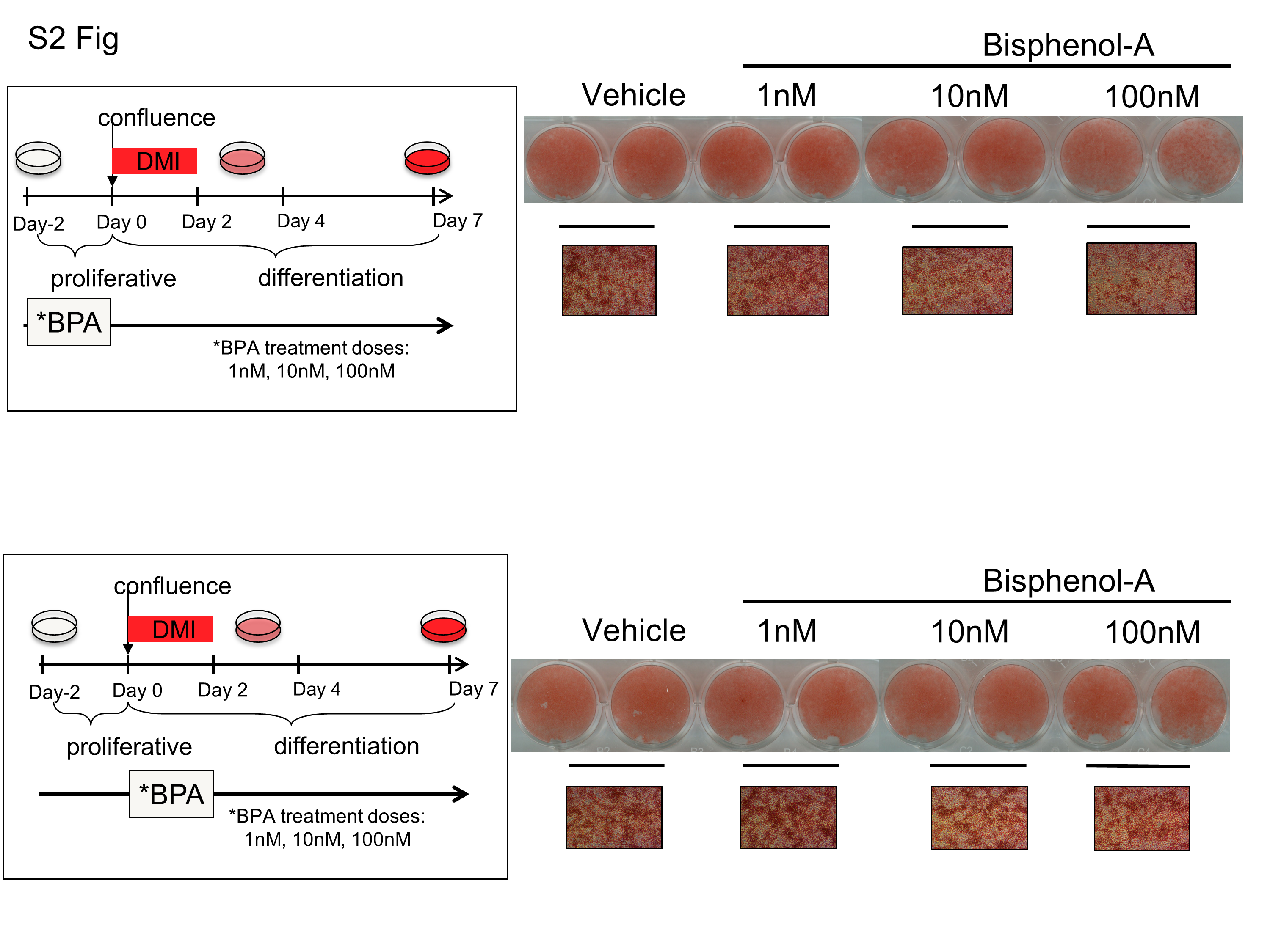

Supplement: S2 Fig — Confluent 3T3-L1 were exposed to several concentrations of BPA (1-100nM) or vehicle (0.1% ethanol) from day -2 to day 0 or from day 0 to day 2. Adipocyte differentiation was induced at day 0 with media enriched with DMI. (Left Panels) Schematic representation of the treatment concentrations and time course applied. (Right Panels) Triglyceride accumulation visualized by oil Red O staining and representative bright field microscopy images (40X magnification) were acquired between day 8–9. (TIF) [file pone.0201122.s002.TIF]

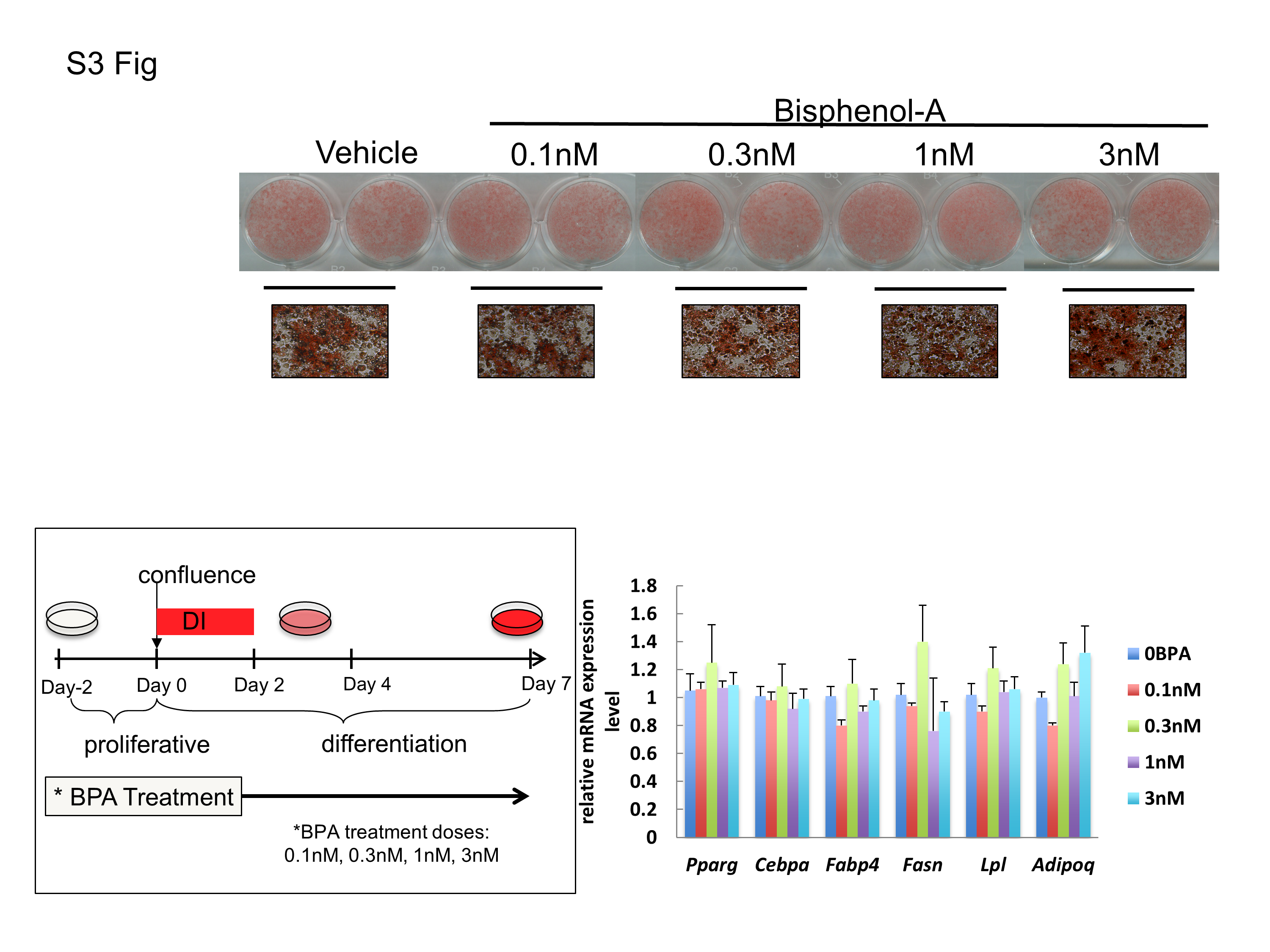

Supplement: S3 Fig — Confluent 3T3-L1 cells were exposed to several doses of BPA (0.1-3nM) or vehicle (0.1% ethanol) from day -2 to day 2. Suboptimal adipocyte differentiation was induced at day 0 with media enriched with only with DI (as detailed in the Methods). (A) Schematic representation of the treatment dose and time course applied. (B) Triglyceride accumulation visualized by Oil Red O staining and representative bright field microscopy images (40X magnification) were acquired between day 8–9. (C) Quantitative reverse transcription PCR (qRT-PCR) analysis of adipocyte marker gene expression was completed between day 8–9. Gene expression was normalized to 36B4 level and is presented as relative mRNA expression. (TIF) [file pone.0201122.s003.TIF]

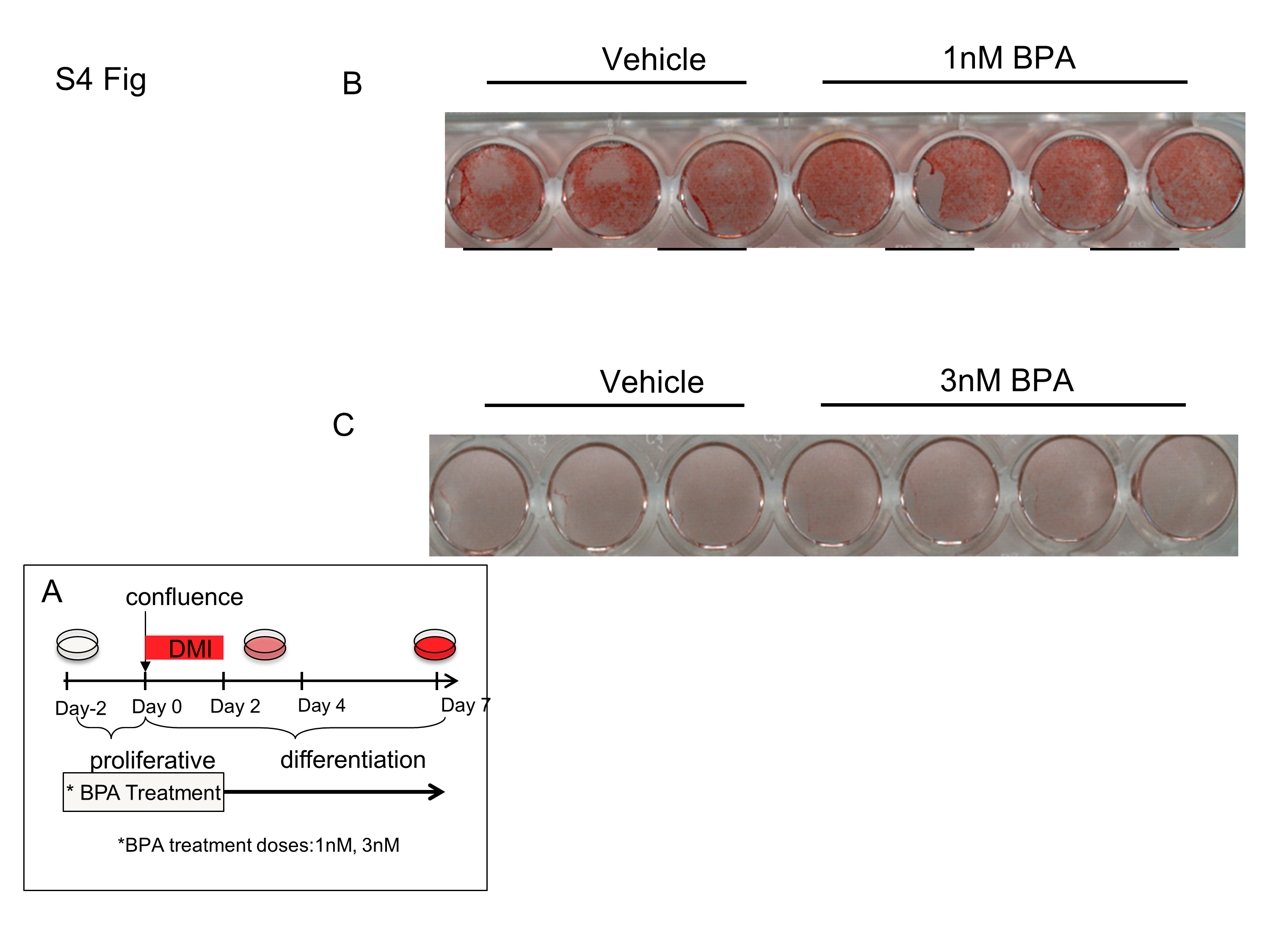

Supplement: S4 Fig — Confluent C3H10T1/2 cells were exposed to either 1nM or 3nM of BPA or vehicle (0.1% ethanol) from day -2 to day 2. Suboptimal adipocyte differentiation was induced at day 0 with media enriched with only with DMI (as detailed in the Methods). (A) Schematic representation of the treatment dose and time course applied. (B & C) Triglyceride accumulation visualized by oil Red O staining and representative bright field microscopy images (40X magnification) were acquired between day 8–9. (TIF) [file pone.0201122.s004.TIF]

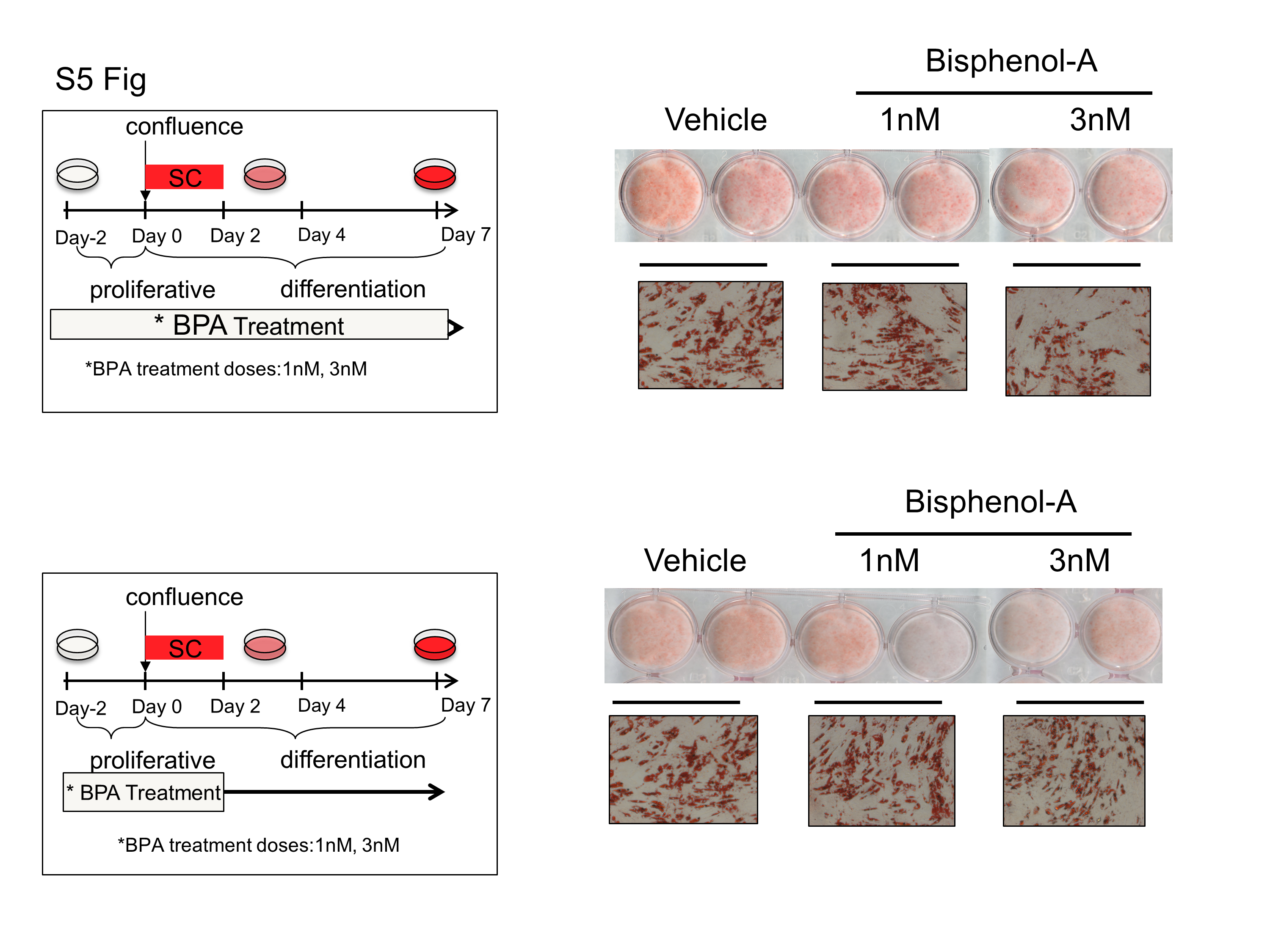

Supplement: S5 Fig — Confluent hADSCs were exposed to several concentrations of BPA (1-3nM) or vehicle (0.1% ethanol) from day -2 to day 7 or from day -2 to day 2. Adipocyte differentiation was induced at day 0 with media enriched with suboptimal conditions (SC) (as detailed in the Methods section). (Left Panels) Schematic representation of the treatment concentrations and time course applied. (Right Panels) Triglyceride accumulation visualized by oil Red O staining and representative bright field microscopy images (40X magnification) were acquired between day 8–9. (TIF) [file pone.0201122.s005.TIF]
